# *N* ^1^-(Thio­phen-2-ylmeth­yl)-*N* ^3^,*N* ^3^-bis­[3-(thio­phen-2-yl­methyl­ammonio)­prop­yl]propane-1,3-di­ammonium hexa­fluorido­silicate methanol tris­olvate

**DOI:** 10.1107/S1600536813029565

**Published:** 2013-11-06

**Authors:** Syed A. Haque, Dominique N. Cooper, Douglas R. Powell, Ramaiyer Venkatraman, Md. Alamgir Hossain

**Affiliations:** a Department of Chemistry and Biochemistry, Jackson State University, Jackson, MS 39217, USA; b Department of Chemistry and Biochemistry, University of Oklahoma, Norman, OK 73019, USA

## Abstract

In the title compound, C_24_H_40_N_4_S_3_^4+^·2SiF_6_^2−^·3CH_3_OH, the central tertiary amine function is protonated and is connected to three thio­phen-2-yl­methyl­amino-*n*-propyl groups, forming the arms of a T-shaped cation that has two pockets. Each arm contains one protonated secondary amine function, and each pocket is occupied by one SiF_6_^2−^ anion bonded *via* two N—H⋯F inter­actions with the protonated amine group on the middle arm, while two methanol solvent mol­ecules are N—H⋯O hydrogen-bonded with the other secondary protonated amine groups on the side arms. Weak O—H⋯O and O—H⋯F hydrogen bonds between the solvent mol­ecules and between the solvent mol­ecules and the anions, respectively, are also observed. All three thio­phene groups in the arms are disordered over two sets of sites, with occupancy ratios of 0.828 (3):0.172 (3), 0.910 (2):0.090 (2) and 0.890 (3):0.110 (3).

## Related literature

For background to polyamine-based mol­ecules, see: McKee *et al.* (2003 ▶); Hossain (2008 ▶); Mendy *et al.* (2010 ▶). For our previous work on this class of compound, see: Işıklan *et al.* (2011 ▶); Hossain *et al.* (2011 ▶). For related structures, see: Hossain *et al.* (2012 ▶); Pilate *et al.* (2010 ▶).
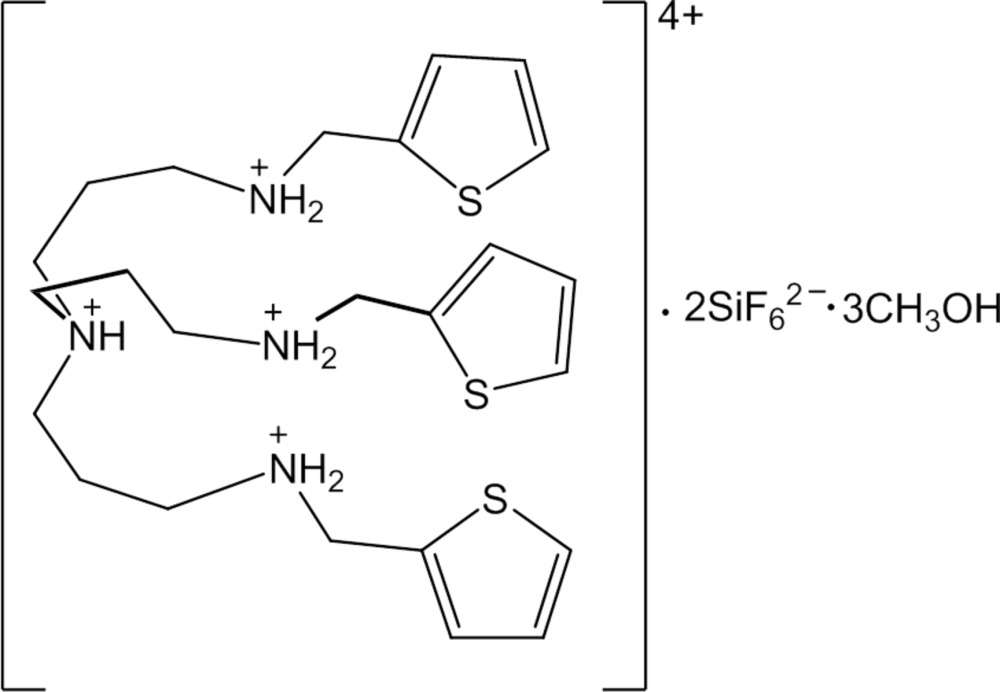


## Experimental

### 

#### Crystal data

C_24_H_40_N_4_S_3_^4+^·2SiF_6_^2−^·3CH_4_O
*M* *_r_* = 861.09
Triclinic, 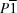

*a* = 8.4854 (4) Å
*b* = 12.5107 (6) Å
*c* = 18.9003 (10) Å
α = 89.024 (2)°
β = 87.750 (2)°
γ = 72.206 (3)°
*V* = 1908.94 (16) Å^3^
*Z* = 2
Mo *K*α radiation
μ = 0.35 mm^−1^
*T* = 100 K
0.58 × 0.16 × 0.03 mm

#### Data collection

Bruker APEX CCD diffractometer
Absorption correction: multi-scan (*SADABS*; Bruker, 2002 ▶) *T*_min_ = 0.822, *T*_max_ = 0.990
25975 measured reflections
9447 independent reflections
7815 reflections with *I* > 2σ(*I*)
*R*_int_ = 0.021

#### Refinement

*R*[*F* ^2^ > 2σ(*F* ^2^)] = 0.044
*wR*(*F* ^2^) = 0.125
*S* = 1.00
9447 reflections
604 parameters
642 restraints
H atoms treated by a mixture of independent and constrained refinement
Δρ_max_ = 0.94 e Å^−3^
Δρ_min_ = −0.80 e Å^−3^

### 

Data collection: *SMART* (Bruker, 2007 ▶); cell refinement: *SAINT* (Bruker, 2007 ▶); data reduction: *SAINT*; program(s) used to solve structure: *SHELXTL* (Sheldrick, 2008 ▶); program(s) used to refine structure: *SHELXTL*; molecular graphics: *SHELXTL*; software used to prepare material for publication: *SHELXTL*.

## Supplementary Material

Crystal structure: contains datablock(s) global, I. DOI: 10.1107/S1600536813029565/wm2779sup1.cif

Structure factors: contains datablock(s) I. DOI: 10.1107/S1600536813029565/wm2779Isup2.hkl

Additional supplementary materials:  crystallographic information; 3D view; checkCIF report

## Figures and Tables

**Table 1:** Hydrogen-bond geometry (Å, °)

*D*—H⋯*A*	*D*—H	H⋯*A*	*D*⋯*A*	*D*—H⋯*A*
N1—H1⋯F4*D* ^i^	0.90 (3)	2.51 (2)	3.171 (2)	131.3 (18)
N1—H1⋯F5*D* ^i^	0.90 (3)	2.54 (2)	3.2005 (19)	131.1 (19)
N1—H1⋯F6*D* ^i^	0.90 (2)	1.91 (2)	2.7810 (18)	162 (2)
N5*A*—H5*A*1⋯F1*D*	0.82 (2)	2.02 (2)	2.775 (2)	153 (2)
N5*A*—H5*A*1⋯F6*D*	0.82 (2)	2.44 (2)	2.985 (2)	125 (2)
N5*A*—H5*A*2⋯F3*E*	0.83 (2)	1.94 (2)	2.759 (2)	172 (2)
N5*A*—H5*A*2⋯F4*E*	0.83 (2)	2.63 (2)	3.029 (2)	111 (2)
N5*B*—H5*B*1⋯F1*E* ^ii^	0.82 (2)	1.92 (2)	2.736 (2)	170 (2)
N5*B*—H5*B*1⋯F2*E* ^ii^	0.82 (2)	2.47 (2)	2.969 (2)	120 (2)
N5*B*—H5*B*2⋯O1*F*	0.84 (2)	1.98 (2)	2.790 (2)	163 (2)
N5*C*—H5*C*1⋯F5*E* ^i^	0.84 (2)	2.00 (2)	2.828 (2)	171 (3)
N5*C*—H5*C*1⋯F1*E* ^i^	0.84 (2)	2.41 (2)	2.932 (2)	121 (2)
N5*C*—H5*C*2⋯O3*F*	0.86 (2)	1.92 (2)	2.758 (3)	168 (3)
O1*F*—H1*F*⋯F3*D* ^i^	0.84	1.95	2.7374 (19)	156
O1*F*—H1*F*⋯F4*D* ^i^	0.84	2.43	3.144 (2)	139
O3*F*—H3*F*⋯F6*E*	0.84	2.13	2.974 (3)	180
O3*F*—H3*F*⋯F4*E*	0.84	2.61	3.118 (3)	120
O5*F*—H5*F*⋯O3*F*	0.84	2.39	3.226 (5)	179

Symmetry codes: (i) 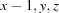; (ii) 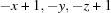.

**Fig. 1.**
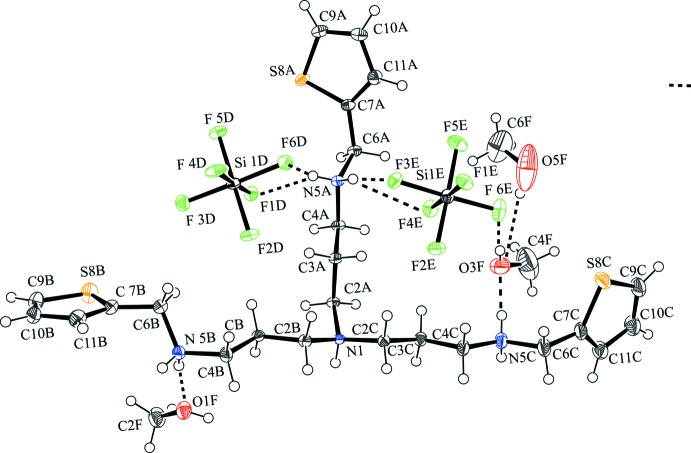
The molecular entities of the title compound, showing the atom-numbering scheme and hydrogen bonding interactions (only the major disordered parts of the thiophene rings are shown). Displacement ellipsoids are drawn at the 50% probability level.

**Fig. 2.**
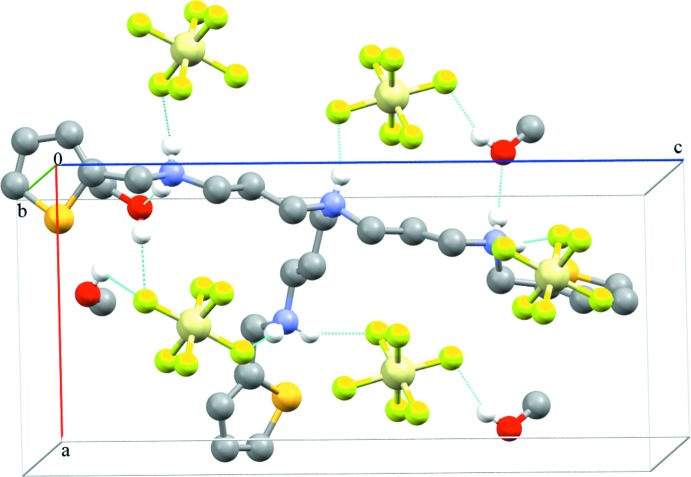
The expanded content of the unit cell of the title compound as viewed along the b axis, showing all interacting hexafluoridosilicate anions with the cation and the methanol solvent molecules. Only major hydrogen bonding interactions are shown for clarity. A full list of hydrogen bonds is listed in Table 1.

## supplementary crystallographic information

### 1. Comment

Polyamine-based synthetic molecules are known as excellent hosts for binding to a variety of anions both in solution and solid state (McKee *et al.*, 2003). Such molecules are particularly useful for selective recognition of environmentally and biologically important polyatomic anions, forming stable host-guest complexes *via* hydrogen bonding and electrostatic interactions (Hossain, 2008; Mendy *et al.*, 2010). For example, our group recently reported a tripodal host containing ethylene chains in the arms, which provides the complementary of binding sites for a *C_3_*-symmetric nitrate anion (Işıklan *et al.*, 2011). A related tripodal host was found to fully encapsulate a dihydrogen phosphate anion within its cavity (Hossain *et al.*, 2011). In order to obtain the flexible cavity for polyatomic anions, we were interested in synthesizing a tripodal molecule with larger propylene chains. Herein, we report the crystal structure of the title compound, (C_24_H_40_N_4_S_3_)[SiF_6_]_2_^.^3(CH_3_OH), that contains two hexafluoridosilicate anions (SiF_6_^2-^) which originated from the glass container during the crystallization process of the fluoride salt of the ligand. The formation of SiF_6_^2-^ was previously observed by us for a large macrocyclic-based receptor forming a water-fluoride cluster in a molecular box (Hossain *et al.*, 2012).

The cation of the title compound adopts a T-shaped geometry rather than a commonly observed tripodal pocket (Pilate *et al.*, 2010). All four nitrogen atoms in the amine groups are protonated, and the charges are balanced by two dinegatively charged hexafluoridosilicate anions. As shown in Fig. 1, the cationic unit contains two pockets and each pocket is occupied by a single anion bonded to two NH groups with N—H···F bonds in the range of 2.759 (2) to 3.029 (2) Å (Table 1). The single crystal contains three methanol solvent molecules that are also involved in hydrogen bonding interactions. Two methanol molecules (O1 and O3) are held with two side arms, each connecting with a single N—H···O bond. One (O3) of them is interconnected to the third methanol (O5) *via* one relatively weak O—H···O bond (OH···O = 3.226 (5) Å), and serves also as hydrogen bond donor with two fluoride atoms (F6E and F4E) of a hexafluoridosilicate anion. In the unit cell (Fig. 2), other three nitrogens (N1, N5B and N5C) are also involved in interacting anions via N—H···F bonds (NH···F = 2.758 (3) to 3.2005 (19) Å), each with one SiF_6_^2-^.

### 2. Experimental

Tris(3-aminopropyl)amine (0.80 g,4.24 mmol) was dissolved in 50 mL of diethyl ether in a round bottom flask. To this solution, 2-thiophenealdehyde (1.43 g m, 12.7 mmol) dissolved in 50 mL of EtOH was added and left overnight with constant stirring at room temperature. After completion of the reaction, the solvent was evaporated and the product was diluted with methanol (100 mL). Sodium borohydride (2.0 g) was added to the reaction mixture which was stirred overnight at room temperature. After the evaporation of the solvent, the residue was partitioned in water and CH_2_Cl_2_ (50/50, *v*/*v*). The organic layer was collected and dried with anhydrous MgSO_4_. The solvent was evaporated under reduced pressure to give an oily product of the free amine. Yield 1.38 g (69%). ^1^H NMR (500 MHz, CDCl_3_,): δ 7.20 (d, 3H, *J* = 5.0 Ar*H*), 6.94 (dd, 3H, *J=5.5*,Ar*H*), δ 6.91 (d, 3H, *J* = 3.15 Ar*H*), 3.95 (s, 6H, ArC*H_2_*), 2.65 (t, *J1* = 6.9 Hz,, 6H, Alph*H*), 2.44 (t, *J1* = 7.1 Hz, 6H, Alph*H*), 1.82 (broad s, 3H, N*H*), 1.62 (m, *J1* = 7.1 Hz, 6H, Alph*H*). ^13^C NMR (125 MHz, CDCl_3_,): δ 144.19 (Ar-*C*), 126.52(Ar-*C*), δ 124.75 (Ar-*C*), 124.18(Ar-*C*), 52.24 (Alph-*C*), 48.47(Alph-*C*), 47.79 (Alph-*C*), 27.17 (Alph-*C*), ESI-MS: m/*z* (+) 477.8 [*M* + H]^+^.

The hexafluoride salt was obtained by the dropwise addition of hydrofluoric acid into a glass vial containing free amine (40 mg, 0.08 mmol) in methanol (2 mL) until the pH of the solution became to 2.0. The white precipitate obtained was redissolved in water (1:2, *v/v*, 1 mL) and the crystals suitable for X-ray analysis were grown after five days from slow evaporation of the solvent at room temperature.

### 3. Refinement

H atoms on C were placed in idealized positions with C—H distances 0.95 - 0.99 Å and thereafter treated as riding. The coordinates of those on N were refined; H atoms of the hydroxy function of methanol were found from difference syntheses and were refined with a distance restraint of 0.84 Å. *U*_iso_ for H was assigned as 1.2 times *U*_eq_ of the attached atom (1.5 for methyl). A torsional parameter was refined for each methyl group. The largest residual density peak was 1.50 Å from O2.

The constraints and restraints used in refinement were as follows. The geometry of 1-2 and 1-3 distances of non-hydrogen atoms in the three thiophene groups and the adjacent carbons were restrained to be similar with a standard deviation of 0.004 Å for 1-2 distances and 0.006-0.008 Å for 1-3 distances. In addition, these same atoms were restrained to be approximately in a plane within a standard deviation of 0.008 Å. The displacement parameters of these atoms were restrained to conform to a "rigid bond", i.e., the components of the displacement parameters in the directions of bonds were restrained to be equal within a standard deviation of 0.006 Å. The displacement parameters of the disordered atoms were also restrained to be similar if the atoms were within 2.0 Å of other disordered atoms with a standard deviation of 0.006. The atoms C11M, C11N, and C11O were restrained to have approximately isotropic displacement parameters with a standard deviation of 0.006. The bond lengths of the N5-H groups were all restrainted to be about 0.80Å with a standard deviation of 0.02. Finally the displacement parameters of the disordered pairs of the C7 atoms, that were very close to each other, were constrained to be equal.

### Crystal data

**Table:**

C_24_H_40_N_4_S_3_^4+^·2SiF_6_^2^^−^·3CH_4_O	*Z* = 2
*M**_r_* = 861.09	*F*(000) = 900
Triclinic, *P*1	*D*_x_ = 1.498 Mg m^−^^3^
Hall symbol: -P 1	Mo *K*α radiation, λ = 0.71073 Å
*a* = 8.4854 (4) Å	Cell parameters from 9116 reflections
*b* = 12.5107 (6) Å	θ = 2.5–28.3°
*c* = 18.9003 (10) Å	µ = 0.35 mm^−^^1^
α = 89.024 (2)°	*T* = 100 K
β = 87.750 (2)°	Plate, colorless
γ = 72.206 (3)°	0.58 × 0.16 × 0.03 mm
*V* = 1908.94 (16) Å^3^	

### Data collection

**Table:**

Bruker APEX CCD diffractometer	9447 independent reflections
Radiation source: fine-focus sealed tube	7815 reflections with *I* > 2σ(*I*)
Graphite monochromator	*R*_int_ = 0.021
φ and ω scans	θ_max_ = 28.3°, θ_min_ = 1.7°
Absorption correction: multi-scan (*SADABS*; Bruker, 2002)	*h* = −11→11
*T*_min_ = 0.822, *T*_max_ = 0.990	*k* = −16→16
25975 measured reflections	*l* = −24→25

### Refinement

**Table:**

Refinement on *F*^2^	Primary atom site location: structure-invariant direct methods
Least-squares matrix: full	Secondary atom site location: difference Fourier map
*R*[*F*^2^ > 2σ(*F*^2^)] = 0.044	Hydrogen site location: inferred from neighbouring sites
*wR*(*F*^2^) = 0.125	H atoms treated by a mixture of independent and constrained refinement
*S* = 1.00	*w* = 1/[σ^2^(*F*_o_^2^) + (0.070*P*)^2^ + 1.560*P*] where *P* = (*F*_o_^2^ + 2*F*_c_^2^)/3
9447 reflections	(Δ/σ)_max_ = 0.006
604 parameters	Δρ_max_ = 0.94 e Å^−^^3^
642 restraints	Δρ_min_ = −0.80 e Å^−^^3^

### Fractional atomic coordinates and isotropic or equivalent isotropic displacement parameters (Å^2^)

**Table:**

	*x*	*y*	*z*	*U*_iso_*/*U*_eq_	Occ. (<1)
N1	0.13157 (18)	0.16663 (12)	0.44971 (8)	0.0129 (3)	
H1	0.025 (3)	0.1828 (19)	0.4645 (12)	0.015*	
C2A	0.1602 (2)	0.27924 (14)	0.44162 (10)	0.0143 (3)	
H2A1	0.1293	0.3205	0.4868	0.017*	
H2A2	0.0876	0.3233	0.4048	0.017*	
C3A	0.3400 (2)	0.26928 (15)	0.42114 (10)	0.0159 (3)	
H3A1	0.4114	0.2399	0.4617	0.019*	
H3A2	0.3790	0.2166	0.3811	0.019*	
C4A	0.3505 (2)	0.38465 (15)	0.39995 (10)	0.0159 (3)	
H4A1	0.2840	0.4119	0.3577	0.019*	
H4A2	0.3044	0.4382	0.4390	0.019*	
N5A	0.52685 (19)	0.37908 (13)	0.38383 (9)	0.0153 (3)	
H5A1	0.570 (3)	0.376 (2)	0.4221 (10)	0.018*	
H5A2	0.576 (3)	0.3214 (16)	0.3613 (11)	0.018*	
C6A	0.53771 (19)	0.47655 (14)	0.34078 (9)	0.0168 (3)	
H6A1	0.4614	0.5456	0.3623	0.020*	
H6A2	0.4985	0.4697	0.2929	0.020*	
C7A	0.7067 (2)	0.49026 (14)	0.33317 (11)	0.0174 (3)	0.828 (3)
S8A	0.77713 (7)	0.56389 (5)	0.39491 (3)	0.01999 (19)	0.828 (3)
C9A	0.9620 (3)	0.5433 (2)	0.34880 (15)	0.0206 (5)	0.828 (3)
H9A	1.0477	0.5719	0.3627	0.025*	0.828 (3)
C10A	0.9704 (3)	0.4808 (2)	0.28972 (15)	0.0222 (5)	0.828 (3)
H10A	1.0636	0.4598	0.2576	0.027*	0.828 (3)
C11A	0.8239 (4)	0.4505 (3)	0.28160 (17)	0.0210 (7)	0.828 (3)
H11A	0.8097	0.4064	0.2434	0.025*	0.828 (3)
C7A'	0.7048 (4)	0.4918 (4)	0.3297 (3)	0.0174 (3)	0.172 (3)
S8A'	0.8363 (4)	0.4256 (4)	0.2610 (2)	0.0204 (9)	0.172 (3)
C9A'	0.9851 (9)	0.4837 (8)	0.2850 (6)	0.0183 (15)	0.172 (3)
H9A'	1.0894	0.4708	0.2608	0.022*	0.172 (3)
C10M	0.9351 (12)	0.5504 (11)	0.3430 (7)	0.0186 (14)	0.172 (3)
H10M	1.0007	0.5898	0.3640	0.022*	0.172 (3)
C11M	0.7739 (10)	0.5543 (9)	0.3684 (5)	0.0202 (13)	0.172 (3)
H11M	0.7201	0.5965	0.4085	0.024*	0.172 (3)
C2B	0.2348 (2)	0.09184 (15)	0.50512 (9)	0.0154 (3)	
H2B1	0.3507	0.0634	0.4866	0.018*	
H2B2	0.1940	0.0263	0.5133	0.018*	
C3B	0.2323 (2)	0.14949 (15)	0.57565 (9)	0.0145 (3)	
H3B1	0.3094	0.1951	0.5731	0.017*	
H3B2	0.1195	0.1996	0.5874	0.017*	
C4B	0.2852 (2)	0.05860 (16)	0.63168 (10)	0.0192 (4)	
H4B1	0.2107	0.0112	0.6318	0.023*	
H4B2	0.3989	0.0102	0.6197	0.023*	
N5B	0.2812 (2)	0.10644 (14)	0.70420 (9)	0.0183 (3)	
H5B1	0.291 (3)	0.0564 (17)	0.7339 (11)	0.022*	
H5B2	0.186 (2)	0.1499 (18)	0.7114 (13)	0.022*	
C6B	0.4083 (2)	0.16507 (18)	0.71464 (9)	0.0241 (4)	
H6B1	0.5163	0.1190	0.6940	0.029*	
H6B2	0.3757	0.2379	0.6892	0.029*	
C7B	0.42679 (18)	0.18499 (18)	0.79115 (10)	0.0218 (4)	0.910 (2)
S8B	0.36470 (9)	0.31908 (5)	0.82559 (4)	0.03128 (19)	0.910 (2)
C9B	0.4257 (3)	0.27031 (19)	0.90860 (11)	0.0291 (5)	0.910 (2)
H9B	0.4161	0.3169	0.9487	0.035*	0.910 (2)
C10B	0.4896 (3)	0.15638 (18)	0.90968 (11)	0.0249 (5)	0.910 (2)
H10B	0.5300	0.1139	0.9509	0.030*	0.910 (2)
C11B	0.4891 (5)	0.1083 (3)	0.84208 (15)	0.0276 (6)	0.910 (2)
H11B	0.5287	0.0298	0.8336	0.033*	0.910 (2)
C7B'	0.4050 (9)	0.1995 (7)	0.7902 (2)	0.0218 (4)	0.090 (2)
S8B'	0.4924 (16)	0.1029 (9)	0.8549 (3)	0.0307 (16)	0.090 (2)
C9B'	0.438 (2)	0.2041 (13)	0.9192 (5)	0.031 (2)	0.090 (2)
H9B'	0.4622	0.1903	0.9678	0.037*	0.090 (2)
C10N	0.357 (3)	0.3066 (11)	0.8918 (6)	0.0303 (19)	0.090 (2)
H10N	0.3172	0.3730	0.9193	0.036*	0.090 (2)
C11N	0.338 (2)	0.3031 (9)	0.8178 (6)	0.0279 (18)	0.090 (2)
H11N	0.2846	0.3672	0.7903	0.033*	0.090 (2)
C2C	0.1575 (2)	0.10067 (15)	0.38197 (9)	0.0153 (3)	
H2C1	0.1239	0.0324	0.3908	0.018*	
H2C2	0.2772	0.0762	0.3688	0.018*	
C3C	0.0627 (2)	0.16447 (16)	0.31978 (10)	0.0182 (4)	
H3C1	0.1074	0.2259	0.3044	0.022*	
H3C2	−0.0560	0.1973	0.3333	0.022*	
C4C	0.0845 (2)	0.07987 (17)	0.26041 (10)	0.0215 (4)	
H4C1	0.2037	0.0386	0.2533	0.026*	
H4C2	0.0265	0.0247	0.2744	0.026*	
N5C	0.0187 (2)	0.13442 (16)	0.19252 (9)	0.0232 (4)	
H5C1	−0.084 (2)	0.168 (2)	0.1948 (14)	0.028*	
H5C2	0.063 (3)	0.1843 (19)	0.1787 (13)	0.028*	
C6C	0.0497 (3)	0.04944 (17)	0.13373 (9)	0.0272 (4)	
H6C1	0.1660	0.0004	0.1344	0.033*	
H6C2	−0.0233	0.0017	0.1416	0.033*	
C7C	0.0177 (3)	0.10602 (15)	0.06322 (10)	0.0279 (4)	0.890 (3)
S8C	0.18115 (8)	0.11894 (7)	0.01032 (3)	0.0321 (2)	0.890 (3)
C9C	0.0503 (3)	0.1870 (3)	−0.05466 (13)	0.0354 (6)	0.890 (3)
H9C	0.0857	0.2128	−0.0982	0.042*	0.890 (3)
C10C	−0.1108 (3)	0.1990 (3)	−0.03549 (15)	0.0361 (6)	0.890 (3)
H10C	−0.2017	0.2351	−0.0641	0.043*	0.890 (3)
C11C	−0.1271 (4)	0.1517 (3)	0.03181 (16)	0.0329 (7)	0.890 (3)
H11C	−0.2308	0.1521	0.0529	0.039*	0.890 (3)
C7C'	0.0154 (9)	0.1106 (7)	0.0647 (3)	0.0279 (4)	0.110 (3)
S8C'	−0.1832 (8)	0.1641 (9)	0.0348 (4)	0.0447 (16)	0.110 (3)
C9C'	−0.1166 (18)	0.2163 (16)	−0.0414 (6)	0.0363 (18)	0.110 (3)
H9C'	−0.1874	0.2559	−0.0772	0.044*	0.110 (3)
C10O	0.0513 (18)	0.194 (2)	−0.0437 (8)	0.0361 (17)	0.110 (3)
H10O	0.1122	0.2153	−0.0815	0.043*	0.110 (3)
C11O	0.1238 (15)	0.1333 (18)	0.0173 (7)	0.0342 (16)	0.110 (3)
H11O	0.2397	0.1108	0.0241	0.041*	0.110 (3)
Si1D	0.73108 (6)	0.23998 (4)	0.55231 (3)	0.01483 (11)	
F1D	0.57427 (13)	0.35369 (9)	0.52832 (6)	0.0184 (2)	
F2D	0.60821 (13)	0.15817 (9)	0.54819 (7)	0.0226 (2)	
F3D	0.67788 (14)	0.26449 (10)	0.63891 (6)	0.0230 (2)	
F4D	0.89313 (13)	0.12794 (9)	0.57423 (7)	0.0232 (2)	
F5D	0.85612 (13)	0.32209 (9)	0.55567 (6)	0.0211 (2)	
F6D	0.78968 (14)	0.21482 (10)	0.46498 (6)	0.0238 (3)	
Si1E	0.56253 (6)	0.18625 (4)	0.22561 (3)	0.01778 (12)	
F1E	0.71772 (14)	0.06581 (9)	0.20444 (6)	0.0224 (2)	
F2E	0.45671 (14)	0.10710 (10)	0.26609 (7)	0.0239 (3)	
F3E	0.65991 (14)	0.19000 (9)	0.30210 (6)	0.0215 (2)	
F4E	0.41488 (14)	0.30467 (10)	0.24795 (6)	0.0240 (3)	
F5E	0.68021 (16)	0.26028 (10)	0.18600 (7)	0.0290 (3)	
F6E	0.47386 (17)	0.18042 (11)	0.14878 (7)	0.0338 (3)	
O1F	−0.05819 (18)	0.21010 (13)	0.72577 (8)	0.0281 (3)	
H1F	−0.1174	0.2165	0.6904	0.034*	
C2F	−0.1528 (3)	0.2917 (2)	0.77516 (13)	0.0383 (6)	
H2F1	−0.2085	0.3613	0.7499	0.058*	
H2F2	−0.0793	0.3066	0.8098	0.058*	
H2F3	−0.2359	0.2632	0.7997	0.058*	
O3F	0.1189 (3)	0.3171 (2)	0.15212 (11)	0.0542 (6)	
H3F	0.2191	0.2785	0.1512	0.065*	
C4F	0.0257 (8)	0.3911 (4)	0.0983 (2)	0.0957 (17)	
H4F1	0.0258	0.4681	0.1069	0.144*	
H4F2	−0.0886	0.3884	0.1000	0.144*	
H4F3	0.0768	0.3669	0.0515	0.144*	
O5F	0.4231 (5)	0.3749 (2)	0.06952 (15)	0.1084 (13)	
H5F	0.3443	0.3593	0.0911	0.130*	
C6F	0.4393 (6)	0.4698 (3)	0.0981 (2)	0.0769 (12)	
H6F1	0.4004	0.4753	0.1478	0.115*	
H6F2	0.3733	0.5354	0.0719	0.115*	
H6F3	0.5560	0.4671	0.0952	0.115*	

### Atomic displacement parameters (Å^2^)

**Table:**

	*U*^11^	*U*^22^	*U*^33^	*U*^12^	*U*^13^	*U*^23^
N1	0.0116 (7)	0.0140 (7)	0.0140 (7)	−0.0053 (5)	0.0001 (5)	0.0006 (5)
C2A	0.0129 (8)	0.0126 (8)	0.0181 (8)	−0.0052 (6)	−0.0003 (6)	0.0011 (6)
C3A	0.0117 (8)	0.0170 (8)	0.0195 (9)	−0.0054 (6)	0.0000 (6)	0.0011 (7)
C4A	0.0116 (8)	0.0163 (8)	0.0204 (9)	−0.0055 (6)	0.0007 (6)	−0.0005 (7)
N5A	0.0135 (7)	0.0151 (7)	0.0182 (8)	−0.0059 (6)	−0.0005 (6)	0.0010 (6)
C6A	0.0147 (8)	0.0148 (8)	0.0220 (9)	−0.0062 (6)	−0.0008 (7)	0.0015 (7)
C7A	0.0173 (8)	0.0154 (8)	0.0211 (9)	−0.0076 (6)	−0.0002 (7)	0.0004 (6)
S8A	0.0173 (3)	0.0193 (3)	0.0252 (4)	−0.0080 (2)	0.0006 (2)	−0.0068 (2)
C9A	0.0147 (11)	0.0185 (11)	0.0296 (12)	−0.0066 (9)	−0.0027 (9)	0.0026 (9)
C10A	0.0169 (10)	0.0216 (11)	0.0279 (12)	−0.0062 (9)	0.0027 (9)	0.0018 (10)
C11A	0.0217 (12)	0.0180 (15)	0.0233 (15)	−0.0061 (10)	0.0001 (11)	−0.0031 (11)
C7A'	0.0173 (8)	0.0154 (8)	0.0211 (9)	−0.0076 (6)	−0.0002 (7)	0.0004 (6)
S8A'	0.0157 (14)	0.0161 (17)	0.029 (2)	−0.0055 (12)	0.0051 (12)	−0.0014 (13)
C9A'	0.016 (3)	0.017 (3)	0.025 (3)	−0.009 (2)	−0.001 (2)	0.002 (3)
C10M	0.014 (2)	0.017 (3)	0.026 (3)	−0.005 (2)	−0.006 (2)	0.002 (2)
C11M	0.017 (2)	0.018 (2)	0.024 (2)	−0.004 (2)	0.000 (2)	−0.001 (2)
C2B	0.0145 (8)	0.0142 (8)	0.0167 (8)	−0.0032 (6)	−0.0018 (6)	0.0026 (6)
C3B	0.0124 (8)	0.0155 (8)	0.0163 (8)	−0.0052 (6)	−0.0016 (6)	0.0021 (6)
C4B	0.0221 (9)	0.0190 (9)	0.0162 (9)	−0.0057 (7)	−0.0029 (7)	0.0013 (7)
N5B	0.0181 (8)	0.0215 (8)	0.0155 (8)	−0.0064 (6)	−0.0020 (6)	0.0023 (6)
C6B	0.0241 (9)	0.0320 (10)	0.0207 (9)	−0.0151 (8)	−0.0031 (7)	0.0019 (8)
C7B	0.0208 (9)	0.0244 (9)	0.0220 (9)	−0.0093 (7)	−0.0038 (7)	−0.0003 (7)
S8B	0.0416 (4)	0.0203 (3)	0.0307 (3)	−0.0069 (2)	−0.0068 (3)	0.0002 (2)
C9B	0.0341 (13)	0.0312 (13)	0.0232 (11)	−0.0109 (10)	−0.0038 (9)	−0.0060 (9)
C10B	0.0233 (11)	0.0305 (12)	0.0213 (10)	−0.0083 (9)	−0.0042 (8)	0.0012 (9)
C11B	0.0261 (13)	0.0284 (13)	0.0284 (13)	−0.0081 (11)	−0.0024 (12)	−0.0008 (11)
C7B'	0.0208 (9)	0.0244 (9)	0.0220 (9)	−0.0093 (7)	−0.0038 (7)	−0.0003 (7)
S8B'	0.027 (3)	0.031 (2)	0.030 (3)	−0.004 (2)	−0.002 (2)	0.007 (2)
C9B'	0.033 (4)	0.033 (4)	0.024 (3)	−0.007 (4)	−0.006 (3)	0.000 (3)
C10N	0.037 (3)	0.028 (3)	0.027 (3)	−0.011 (3)	−0.006 (3)	−0.002 (3)
C11N	0.032 (3)	0.026 (3)	0.026 (3)	−0.009 (3)	−0.005 (3)	0.001 (2)
C2C	0.0168 (8)	0.0152 (8)	0.0147 (8)	−0.0060 (6)	−0.0008 (6)	−0.0010 (6)
C3C	0.0160 (8)	0.0209 (9)	0.0172 (9)	−0.0046 (7)	−0.0013 (7)	−0.0015 (7)
C4C	0.0201 (9)	0.0276 (10)	0.0164 (9)	−0.0064 (8)	−0.0032 (7)	−0.0023 (7)
N5C	0.0180 (8)	0.0329 (10)	0.0173 (8)	−0.0053 (7)	−0.0015 (6)	−0.0040 (7)
C6C	0.0242 (10)	0.0363 (11)	0.0187 (9)	−0.0057 (8)	0.0021 (7)	−0.0080 (8)
C7C	0.0251 (9)	0.0404 (11)	0.0177 (9)	−0.0094 (8)	0.0019 (7)	−0.0081 (8)
S8C	0.0222 (3)	0.0516 (4)	0.0213 (3)	−0.0101 (3)	0.0023 (2)	−0.0001 (3)
C9C	0.0369 (13)	0.0499 (15)	0.0197 (12)	−0.0138 (11)	−0.0029 (10)	0.0025 (12)
C10C	0.0327 (12)	0.0470 (15)	0.0260 (12)	−0.0078 (11)	−0.0044 (10)	−0.0015 (10)
C11C	0.0235 (13)	0.0476 (16)	0.0262 (12)	−0.0089 (14)	0.0037 (11)	−0.0081 (11)
C7C'	0.0251 (9)	0.0404 (11)	0.0177 (9)	−0.0094 (8)	0.0019 (7)	−0.0081 (8)
S8C'	0.040 (3)	0.062 (3)	0.030 (3)	−0.012 (3)	0.000 (2)	−0.002 (2)
C9C'	0.033 (3)	0.048 (3)	0.025 (3)	−0.009 (3)	−0.001 (3)	−0.003 (3)
C10O	0.034 (3)	0.049 (3)	0.024 (3)	−0.011 (3)	−0.001 (3)	−0.002 (3)
C11O	0.032 (2)	0.046 (3)	0.024 (2)	−0.010 (2)	0.001 (2)	−0.004 (2)
Si1D	0.0105 (2)	0.0138 (2)	0.0202 (2)	−0.00363 (17)	−0.00076 (18)	−0.00068 (18)
F1D	0.0143 (5)	0.0182 (5)	0.0197 (5)	−0.0002 (4)	−0.0024 (4)	0.0001 (4)
F2D	0.0134 (5)	0.0209 (6)	0.0354 (7)	−0.0081 (4)	−0.0008 (5)	−0.0018 (5)
F3D	0.0214 (6)	0.0266 (6)	0.0192 (6)	−0.0049 (5)	−0.0018 (4)	0.0019 (4)
F4D	0.0123 (5)	0.0153 (5)	0.0418 (7)	−0.0038 (4)	−0.0034 (5)	0.0032 (5)
F5D	0.0171 (5)	0.0163 (5)	0.0321 (6)	−0.0079 (4)	−0.0047 (5)	0.0016 (4)
F6D	0.0163 (5)	0.0310 (6)	0.0225 (6)	−0.0052 (5)	0.0036 (4)	−0.0076 (5)
Si1E	0.0178 (2)	0.0174 (2)	0.0170 (2)	−0.00358 (19)	−0.00032 (19)	−0.00119 (18)
F1E	0.0222 (6)	0.0178 (5)	0.0247 (6)	−0.0032 (4)	0.0058 (4)	−0.0017 (4)
F2E	0.0188 (5)	0.0229 (6)	0.0315 (6)	−0.0091 (5)	0.0042 (5)	−0.0045 (5)
F3E	0.0189 (5)	0.0216 (6)	0.0232 (6)	−0.0045 (4)	−0.0051 (4)	−0.0025 (4)
F4E	0.0215 (6)	0.0205 (6)	0.0258 (6)	0.0003 (4)	−0.0015 (5)	−0.0035 (5)
F5E	0.0316 (7)	0.0213 (6)	0.0321 (7)	−0.0065 (5)	0.0076 (5)	0.0047 (5)
F6E	0.0382 (7)	0.0352 (7)	0.0234 (6)	−0.0030 (6)	−0.0099 (5)	−0.0060 (5)
O1F	0.0200 (7)	0.0365 (8)	0.0248 (7)	−0.0038 (6)	−0.0038 (6)	−0.0003 (6)
C2F	0.0308 (12)	0.0528 (16)	0.0279 (12)	−0.0077 (11)	0.0009 (9)	−0.0025 (11)
O3F	0.0499 (12)	0.0736 (15)	0.0520 (12)	−0.0392 (11)	0.0187 (10)	−0.0245 (11)
C4F	0.186 (6)	0.068 (3)	0.047 (2)	−0.061 (3)	0.014 (3)	−0.0029 (19)
O5F	0.187 (4)	0.0490 (15)	0.0632 (17)	0.0094 (18)	−0.056 (2)	−0.0066 (13)
C6F	0.121 (4)	0.0403 (18)	0.075 (3)	−0.029 (2)	−0.036 (2)	0.0161 (17)

### Geometric parameters (Å, º)

**Table:**

N1—C2A	1.505 (2)	C9B'—H9B'	0.9500
N1—C2C	1.507 (2)	C10N—C11N	1.418 (4)
N1—C2B	1.509 (2)	C10N—H10N	0.9500
N1—H1	0.90 (2)	C11N—H11N	0.9500
C2A—C3A	1.527 (2)	C2C—C3C	1.522 (3)
C2A—H2A1	0.9900	C2C—H2C1	0.9900
C2A—H2A2	0.9900	C2C—H2C2	0.9900
C3A—C4A	1.519 (2)	C3C—C4C	1.522 (3)
C3A—H3A1	0.9900	C3C—H3C1	0.9900
C3A—H3A2	0.9900	C3C—H3C2	0.9900
C4A—N5A	1.496 (2)	C4C—N5C	1.489 (3)
C4A—H4A1	0.9900	C4C—H4C1	0.9900
C4A—H4A2	0.9900	C4C—H4C2	0.9900
N5A—C6A	1.479 (2)	N5C—C6C	1.510 (2)
N5A—H5A1	0.819 (16)	N5C—H5C1	0.840 (16)
N5A—H5A2	0.829 (16)	N5C—H5C2	0.855 (17)
C6A—C7A'	1.494 (3)	C6C—C7C	1.493 (2)
C6A—C7A	1.497 (2)	C6C—C7C'	1.494 (3)
C6A—H6A1	0.9900	C6C—H6C1	0.9900
C6A—H6A2	0.9900	C6C—H6C2	0.9900
C7A—C11A	1.351 (3)	C7C—C11C	1.343 (3)
C7A—S8A	1.731 (2)	C7C—S8C	1.724 (2)
S8A—C9A	1.715 (2)	S8C—C9C	1.720 (2)
C9A—C10A	1.362 (3)	C9C—C10C	1.364 (3)
C9A—H9A	0.9500	C9C—H9C	0.9500
C10A—C11A	1.420 (3)	C10C—C11C	1.414 (3)
C10A—H10A	0.9500	C10C—H10C	0.9500
C11A—H11A	0.9500	C11C—H11C	0.9500
C7A'—C11M	1.350 (4)	C7C'—C11O	1.348 (4)
C7A'—S8A'	1.725 (4)	C7C'—S8C'	1.726 (4)
S8A'—C9A'	1.716 (4)	S8C'—C9C'	1.716 (4)
C9A'—C10M	1.362 (4)	C9C'—C10O	1.364 (4)
C9A'—H9A'	0.9500	C9C'—H9C'	0.9500
C10M—C11M	1.419 (4)	C10O—C11O	1.420 (4)
C10M—H10M	0.9500	C10O—H10O	0.9500
C11M—H11M	0.9500	C11O—H11O	0.9500
C2B—C3B	1.523 (2)	Si1D—F2D	1.6740 (12)
C2B—H2B1	0.9900	Si1D—F3D	1.6886 (12)
C2B—H2B2	0.9900	Si1D—F5D	1.6908 (12)
C3B—C4B	1.517 (2)	Si1D—F1D	1.6935 (11)
C3B—H3B1	0.9900	Si1D—F4D	1.6954 (12)
C3B—H3B2	0.9900	Si1D—F6D	1.7112 (12)
C4B—N5B	1.500 (2)	Si1E—F4E	1.6698 (12)
C4B—H4B1	0.9900	Si1E—F6E	1.6742 (13)
C4B—H4B2	0.9900	Si1E—F2E	1.6841 (13)
N5B—C6B	1.499 (2)	Si1E—F3E	1.7013 (12)
N5B—H5B1	0.822 (16)	Si1E—F5E	1.7024 (14)
N5B—H5B2	0.835 (16)	Si1E—F1E	1.7120 (12)
C6B—C7B	1.494 (2)	O1F—C2F	1.425 (3)
C6B—C7B'	1.495 (3)	O1F—H1F	0.8401
C6B—H6B1	0.9900	C2F—H2F1	0.9800
C6B—H6B2	0.9900	C2F—H2F2	0.9800
C7B—C11B	1.351 (3)	C2F—H2F3	0.9800
C7B—S8B	1.728 (2)	O3F—C4F	1.450 (5)
S8B—C9B	1.714 (2)	O3F—H3F	0.8399
C9B—C10B	1.362 (3)	C4F—H4F1	0.9800
C9B—H9B	0.9500	C4F—H4F2	0.9800
C10B—C11B	1.422 (3)	C4F—H4F3	0.9800
C10B—H10B	0.9500	O5F—C6F	1.360 (4)
C11B—H11B	0.9500	O5F—H5F	0.8399
C7B'—C11N	1.349 (4)	C6F—H6F1	0.9800
C7B'—S8B'	1.727 (4)	C6F—H6F2	0.9800
S8B'—C9B'	1.715 (4)	C6F—H6F3	0.9800
C9B'—C10N	1.362 (4)		
			
C2A—N1—C2C	113.98 (14)	S8B'—C9B'—H9B'	124.2
C2A—N1—C2B	114.05 (13)	C9B'—C10N—C11N	112.3 (4)
C2C—N1—C2B	107.64 (13)	C9B'—C10N—H10N	123.8
C2A—N1—H1	104.5 (14)	C11N—C10N—H10N	123.8
C2C—N1—H1	109.1 (14)	C7B'—C11N—C10N	113.3 (6)
C2B—N1—H1	107.3 (14)	C7B'—C11N—H11N	123.4
N1—C2A—C3A	112.52 (14)	C10N—C11N—H11N	123.4
N1—C2A—H2A1	109.1	N1—C2C—C3C	114.61 (14)
C3A—C2A—H2A1	109.1	N1—C2C—H2C1	108.6
N1—C2A—H2A2	109.1	C3C—C2C—H2C1	108.6
C3A—C2A—H2A2	109.1	N1—C2C—H2C2	108.6
H2A1—C2A—H2A2	107.8	C3C—C2C—H2C2	108.6
C4A—C3A—C2A	108.76 (14)	H2C1—C2C—H2C2	107.6
C4A—C3A—H3A1	109.9	C2C—C3C—C4C	106.64 (15)
C2A—C3A—H3A1	109.9	C2C—C3C—H3C1	110.4
C4A—C3A—H3A2	109.9	C4C—C3C—H3C1	110.4
C2A—C3A—H3A2	109.9	C2C—C3C—H3C2	110.4
H3A1—C3A—H3A2	108.3	C4C—C3C—H3C2	110.4
N5A—C4A—C3A	110.09 (14)	H3C1—C3C—H3C2	108.6
N5A—C4A—H4A1	109.6	N5C—C4C—C3C	112.26 (16)
C3A—C4A—H4A1	109.6	N5C—C4C—H4C1	109.2
N5A—C4A—H4A2	109.6	C3C—C4C—H4C1	109.2
C3A—C4A—H4A2	109.6	N5C—C4C—H4C2	109.2
H4A1—C4A—H4A2	108.2	C3C—C4C—H4C2	109.2
C6A—N5A—C4A	111.26 (14)	H4C1—C4C—H4C2	107.9
C6A—N5A—H5A1	112.5 (17)	C4C—N5C—C6C	111.14 (16)
C4A—N5A—H5A1	106.1 (17)	C4C—N5C—H5C1	113.4 (18)
C6A—N5A—H5A2	107.9 (16)	C6C—N5C—H5C1	107.3 (18)
C4A—N5A—H5A2	110.5 (16)	C4C—N5C—H5C2	112.8 (18)
H5A1—N5A—H5A2	109 (2)	C6C—N5C—H5C2	106.8 (18)
N5A—C6A—C7A'	117.3 (2)	H5C1—N5C—H5C2	105 (2)
N5A—C6A—C7A	115.30 (11)	C7C—C6C—N5C	111.07 (13)
N5A—C6A—H6A1	108.4	C7C'—C6C—N5C	108.7 (4)
C7A'—C6A—H6A1	108.8	C7C—C6C—H6C1	109.4
C7A—C6A—H6A1	108.4	C7C'—C6C—H6C1	110.8
N5A—C6A—H6A2	108.4	N5C—C6C—H6C1	109.4
C7A'—C6A—H6A2	106.0	C7C—C6C—H6C2	109.4
C7A—C6A—H6A2	108.4	C7C'—C6C—H6C2	110.5
H6A1—C6A—H6A2	107.5	N5C—C6C—H6C2	109.4
C11A—C7A—C6A	128.3 (2)	H6C1—C6C—H6C2	108.0
C11A—C7A—S8A	110.39 (17)	C11C—C7C—C6C	129.1 (2)
C6A—C7A—S8A	121.29 (16)	C11C—C7C—S8C	111.06 (18)
C9A—S8A—C7A	92.13 (11)	C6C—C7C—S8C	119.79 (17)
C10A—C9A—S8A	111.36 (18)	C9C—S8C—C7C	91.74 (12)
C10A—C9A—H9A	124.3	C10C—C9C—S8C	111.17 (19)
S8A—C9A—H9A	124.3	C10C—C9C—H9C	124.4
C9A—C10A—C11A	112.2 (2)	S8C—C9C—H9C	124.4
C9A—C10A—H10A	123.9	C9C—C10C—C11C	112.3 (2)
C11A—C10A—H10A	123.9	C9C—C10C—H10C	123.9
C7A—C11A—C10A	113.9 (3)	C11C—C10C—H10C	123.9
C7A—C11A—H11A	123.1	C7C—C11C—C10C	113.8 (3)
C10A—C11A—H11A	123.1	C7C—C11C—H11C	123.1
C11M—C7A'—C6A	127.8 (5)	C10C—C11C—H11C	123.1
C11M—C7A'—S8A'	111.4 (4)	C11O—C7C'—C6C	128.5 (5)
C6A—C7A'—S8A'	120.8 (4)	C11O—C7C'—S8C'	109.7 (6)
C9A'—S8A'—C7A'	91.5 (4)	C6C—C7C'—S8C'	121.8 (4)
C10M—C9A'—S8A'	111.7 (4)	C9C'—S8C'—C7C'	92.7 (4)
C10M—C9A'—H9A'	124.2	C10O—C9C'—S8C'	111.1 (4)
S8A'—C9A'—H9A'	124.2	C10O—C9C'—H9C'	124.4
C9A'—C10M—C11M	112.3 (4)	S8C'—C9C'—H9C'	124.4
C9A'—C10M—H10M	123.9	C9C'—C10O—C11O	111.7 (4)
C11M—C10M—H10M	123.9	C9C'—C10O—H10O	124.1
C7A'—C11M—C10M	113.1 (5)	C11O—C10O—H10O	124.1
C7A'—C11M—H11M	123.4	C7C'—C11O—C10O	114.8 (6)
C10M—C11M—H11M	123.4	C7C'—C11O—H11O	122.6
N1—C2B—C3B	114.10 (14)	C10O—C11O—H11O	122.6
N1—C2B—H2B1	108.7	F2D—Si1D—F3D	90.44 (6)
C3B—C2B—H2B1	108.7	F2D—Si1D—F5D	179.41 (7)
N1—C2B—H2B2	108.7	F3D—Si1D—F5D	90.13 (6)
C3B—C2B—H2B2	108.7	F2D—Si1D—F1D	90.85 (6)
H2B1—C2B—H2B2	107.6	F3D—Si1D—F1D	91.33 (6)
C4B—C3B—C2B	107.59 (14)	F5D—Si1D—F1D	89.29 (6)
C4B—C3B—H3B1	110.2	F2D—Si1D—F4D	90.83 (6)
C2B—C3B—H3B1	110.2	F3D—Si1D—F4D	90.12 (6)
C4B—C3B—H3B2	110.2	F5D—Si1D—F4D	89.02 (6)
C2B—C3B—H3B2	110.2	F1D—Si1D—F4D	177.77 (6)
H3B1—C3B—H3B2	108.5	F2D—Si1D—F6D	90.55 (6)
N5B—C4B—C3B	112.11 (15)	F3D—Si1D—F6D	178.66 (6)
N5B—C4B—H4B1	109.2	F5D—Si1D—F6D	88.88 (6)
C3B—C4B—H4B1	109.2	F1D—Si1D—F6D	89.55 (6)
N5B—C4B—H4B2	109.2	F4D—Si1D—F6D	88.97 (6)
C3B—C4B—H4B2	109.2	F4E—Si1E—F6E	91.42 (7)
H4B1—C4B—H4B2	107.9	F4E—Si1E—F2E	91.77 (6)
C6B—N5B—C4B	114.69 (15)	F6E—Si1E—F2E	91.28 (7)
C6B—N5B—H5B1	110.1 (17)	F4E—Si1E—F3E	90.40 (6)
C4B—N5B—H5B1	109.3 (17)	F6E—Si1E—F3E	177.79 (7)
C6B—N5B—H5B2	111.0 (17)	F2E—Si1E—F3E	89.90 (6)
C4B—N5B—H5B2	106.5 (17)	F4E—Si1E—F5E	91.14 (6)
H5B1—N5B—H5B2	105 (2)	F6E—Si1E—F5E	90.48 (7)
C7B—C6B—N5B	111.93 (12)	F2E—Si1E—F5E	176.57 (7)
C7B'—C6B—N5B	110.4 (3)	F3E—Si1E—F5E	88.24 (7)
C7B—C6B—H6B1	109.2	F4E—Si1E—F1E	178.29 (7)
C7B'—C6B—H6B1	116.9	F6E—Si1E—F1E	90.17 (7)
N5B—C6B—H6B1	109.2	F2E—Si1E—F1E	88.82 (6)
C7B—C6B—H6B2	109.2	F3E—Si1E—F1E	88.00 (6)
C7B'—C6B—H6B2	102.7	F5E—Si1E—F1E	88.22 (6)
N5B—C6B—H6B2	109.2	C2F—O1F—H1F	105.7
H6B1—C6B—H6B2	107.9	O1F—C2F—H2F1	109.5
C11B—C7B—C6B	128.1 (2)	O1F—C2F—H2F2	109.5
C11B—C7B—S8B	110.71 (19)	H2F1—C2F—H2F2	109.5
C6B—C7B—S8B	121.15 (16)	O1F—C2F—H2F3	109.5
C9B—S8B—C7B	92.08 (10)	H2F1—C2F—H2F3	109.5
C10B—C9B—S8B	111.40 (16)	H2F2—C2F—H2F3	109.5
C10B—C9B—H9B	124.3	C4F—O3F—H3F	129.2
S8B—C9B—H9B	124.3	O3F—C4F—H4F1	109.5
C9B—C10B—C11B	112.3 (2)	O3F—C4F—H4F2	109.5
C9B—C10B—H10B	123.9	H4F1—C4F—H4F2	109.5
C11B—C10B—H10B	123.9	O3F—C4F—H4F3	109.5
C7B—C11B—C10B	113.5 (3)	H4F1—C4F—H4F3	109.5
C7B—C11B—H11B	123.2	H4F2—C4F—H4F3	109.5
C10B—C11B—H11B	123.2	C6F—O5F—H5F	108.3
C11N—C7B'—C6B	127.8 (5)	O5F—C6F—H6F1	109.5
C11N—C7B'—S8B'	111.1 (6)	O5F—C6F—H6F2	109.5
C6B—C7B'—S8B'	121.1 (4)	H6F1—C6F—H6F2	109.5
C9B'—S8B'—C7B'	91.7 (4)	O5F—C6F—H6F3	109.5
C10N—C9B'—S8B'	111.6 (4)	H6F1—C6F—H6F3	109.5
C10N—C9B'—H9B'	124.2	H6F2—C6F—H6F3	109.5
			
C2C—N1—C2A—C3A	−64.80 (19)	C6B—C7B—C11B—C10B	179.58 (16)
C2B—N1—C2A—C3A	59.36 (19)	S8B—C7B—C11B—C10B	−0.7 (3)
N1—C2A—C3A—C4A	168.83 (14)	C9B—C10B—C11B—C7B	0.4 (3)
C2A—C3A—C4A—N5A	176.53 (14)	N5B—C6B—C7B'—C11N	−103.1 (7)
C3A—C4A—N5A—C6A	160.71 (15)	N5B—C6B—C7B'—S8B'	76.8 (7)
C4A—N5A—C6A—C7A'	171.0 (3)	C11N—C7B'—S8B'—C9B'	−0.1 (2)
C4A—N5A—C6A—C7A	169.09 (16)	C6B—C7B'—S8B'—C9B'	179.93 (12)
N5A—C6A—C7A—C11A	92.3 (2)	C7B'—S8B'—C9B'—C10N	0.1 (3)
N5A—C6A—C7A—S8A	−87.28 (15)	S8B'—C9B'—C10N—C11N	−0.1 (4)
C11A—C7A—S8A—C9A	1.60 (15)	C6B—C7B'—C11N—C10N	−180.0 (2)
C6A—C7A—S8A—C9A	−178.74 (9)	S8B'—C7B'—C11N—C10N	0.1 (3)
C7A—S8A—C9A—C10A	−1.32 (17)	C9B'—C10N—C11N—C7B'	0.0 (5)
S8A—C9A—C10A—C11A	0.7 (3)	C2A—N1—C2C—C3C	−51.9 (2)
C6A—C7A—C11A—C10A	178.88 (14)	C2B—N1—C2C—C3C	−179.49 (15)
S8A—C7A—C11A—C10A	−1.5 (2)	N1—C2C—C3C—C4C	−171.96 (15)
C9A—C10A—C11A—C7A	0.5 (3)	C2C—C3C—C4C—N5C	−171.57 (15)
N5A—C6A—C7A'—C11M	−93.3 (4)	C3C—C4C—N5C—C6C	177.08 (16)
N5A—C6A—C7A'—S8A'	86.7 (3)	C4C—N5C—C6C—C7C	−167.71 (17)
C11M—C7A'—S8A'—C9A'	−0.2 (2)	C4C—N5C—C6C—C7C'	−167.9 (3)
C6A—C7A'—S8A'—C9A'	179.87 (12)	N5C—C6C—C7C—C11C	−80.6 (2)
C7A'—S8A'—C9A'—C10M	0.0 (3)	N5C—C6C—C7C—S8C	99.45 (16)
S8A'—C9A'—C10M—C11M	0.2 (4)	C11C—C7C—S8C—C9C	0.03 (16)
C6A—C7A'—C11M—C10M	−179.75 (19)	C6C—C7C—S8C—C9C	179.97 (9)
S8A'—C7A'—C11M—C10M	0.3 (3)	C7C—S8C—C9C—C10C	0.36 (18)
C9A'—C10M—C11M—C7A'	−0.3 (4)	S8C—C9C—C10C—C11C	−0.7 (3)
C2A—N1—C2B—C3B	48.1 (2)	C6C—C7C—C11C—C10C	179.66 (14)
C2C—N1—C2B—C3B	175.60 (14)	S8C—C7C—C11C—C10C	−0.4 (2)
N1—C2B—C3B—C4B	159.79 (14)	C9C—C10C—C11C—C7C	0.7 (3)
C2B—C3B—C4B—N5B	−177.89 (15)	N5C—C6C—C7C'—C11O	98.2 (6)
C3B—C4B—N5B—C6B	−68.1 (2)	N5C—C6C—C7C'—S8C'	−81.8 (6)
C4B—N5B—C6B—C7B	−165.62 (16)	C11O—C7C'—S8C'—C9C'	0.0 (2)
C4B—N5B—C6B—C7B'	−174.4 (3)	C6C—C7C'—S8C'—C9C'	179.98 (13)
N5B—C6B—C7B—C11B	67.9 (2)	C7C'—S8C'—C9C'—C10O	0.0 (3)
N5B—C6B—C7B—S8B	−111.77 (16)	S8C'—C9C'—C10O—C11O	−0.1 (4)
C11B—C7B—S8B—C9B	0.61 (17)	C6C—C7C'—C11O—C10O	180.0 (2)
C6B—C7B—S8B—C9B	−179.63 (9)	S8C'—C7C'—C11O—C10O	0.0 (3)
C7B—S8B—C9B—C10B	−0.38 (16)	C9C'—C10O—C11O—C7C'	0.1 (5)
S8B—C9B—C10B—C11B	0.1 (3)		

### Hydrogen-bond geometry (Å, º)

**Table:**

*D*—H···*A*	*D*—H	H···*A*	*D*···*A*	*D*—H···*A*
N1—H1···F4*D*^i^	0.90 (3)	2.51 (2)	3.171 (2)	131.3 (18)
N1—H1···F5*D*^i^	0.90 (3)	2.54 (2)	3.2005 (19)	131.1 (19)
N1—H1···F6*D*^i^	0.90 (2)	1.91 (2)	2.7810 (18)	162 (2)
N5*A*—H5*A*1···F1*D*	0.82 (2)	2.02 (2)	2.775 (2)	153 (2)
N5*A*—H5*A*1···F6*D*	0.82 (2)	2.44 (2)	2.985 (2)	125 (2)
N5*A*—H5*A*2···F3*E*	0.83 (2)	1.94 (2)	2.759 (2)	172 (2)
N5*A*—H5*A*2···F4*E*	0.83 (2)	2.63 (2)	3.029 (2)	111 (2)
N5*B*—H5*B*1···F1*E*^ii^	0.82 (2)	1.92 (2)	2.736 (2)	170 (2)
N5*B*—H5*B*1···F2*E*^ii^	0.82 (2)	2.47 (2)	2.969 (2)	120 (2)
N5*B*—H5*B*2···O1*F*	0.84 (2)	1.98 (2)	2.790 (2)	163 (2)
N5*C*—H5*C*1···F5*E*^i^	0.84 (2)	2.00 (2)	2.828 (2)	171 (3)
N5*C*—H5*C*1···F1*E*^i^	0.84 (2)	2.41 (2)	2.932 (2)	121 (2)
N5*C*—H5*C*2···O3*F*	0.86 (2)	1.92 (2)	2.758 (3)	168 (3)
O1*F*—H1*F*···F3*D*^i^	0.84	1.95	2.7374 (19)	156
O1*F*—H1*F*···F4*D*^i^	0.84	2.43	3.144 (2)	139
O3*F*—H3*F*···F6*E*	0.84	2.13	2.974 (3)	180
O3*F*—H3*F*···F4*E*	0.84	2.61	3.118 (3)	120
O5*F*—H5*F*···O3*F*	0.84	2.39	3.226 (5)	179

Symmetry codes: (i) *x*−1, *y*, *z*; (ii) −*x*+1, −*y*, −*z*+1.

## References

[bb1] Bruker (2002). *SADABS* Bruker AXS, Inc., Madison, Wisconsin, USA.

[bb2] Bruker (2007). *SMART* and *SAINT* Bruker AXS, Inc., Madison, Wisconsin, USA.

[bb3] Hossain, M. A. (2008). *Curr. Org. Chem.* **12**, 1231–1256.

[bb4] Hossain, M. A., Işıklan, M., Pramanik, A., Saeed, M. A. & Fronczek, F. R. (2011). *Cryst. Growth Des.* **12**, 567–571. 10.1021/cg201464k PMC3306550 22435043

[bb5] Hossain, M. A., Saeed, M. A., Pramanik, A., Wong, B. M., Haque, S. A. & Powell, D. R. (2012). *J. Am. Chem. Soc.* **134**, 11892–11895. 10.1021/ja3043855 PMC3601558 22765503

[bb6] Işıklan, M., Saeed, M. A., Pramanik, A., Wong, B. M., Fronczek, F. R. & Hossain, M. A. (2011). *Cryst. Growth Des.* **11**, 959–963. 10.1021/cg2001859 PMC3086375 21552352

[bb7] McKee, V., Nelson, J. & Town, R. M. (2003). *Chem. Soc. Rev*, **32**, 309–325. 10.1039/b200672n 14518184

[bb8] Mendy, J. S., Pilate, M. L., Horne, T., Day, V. W. & Hossain, M. A. (2010). *Chem. Commun.* **46**, 6084–6086. 10.1039/c0cc01699c PMC3056152 20652195

[bb9] Pilate, M. L., Blount, H., Fronczek, F. R. & Hossain, M. A. (2010). *Acta Cryst.* E**66**, o1833–o1834. 10.1107/S1600536810024323 PMC3006998 21588037

[bb10] Sheldrick, G. M. (2008). *Acta Cryst.* A**64**, 112–122. 10.1107/S0108767307043930 18156677

